# A study on fresh product supply chain management decisions considering subsidies and different transaction contracts

**DOI:** 10.1371/journal.pone.0322800

**Published:** 2025-05-29

**Authors:** Yunting Wu, Aimin Zhu, Lijuan Yu, Wenbo Wang

**Affiliations:** School of Management, Shenyang University of Technology, Shenyang, China; Zhengzhou University of Light Industry, CHINA

## Abstract

With the continuous improvement of people’s living standards and the increasing demand for high-quality fresh products, enhancing the quality of fresh products has become an urgent and crucial issue that requires attention. Existing studies have shown that strengthening the preservation capacity of fresh products at the origin can effectively improve product quality. Therefore, this paper starts from the perspective of enhancing the freshness preservation level at the origin, considering providing financial subsidies to the supplier from both the government and the retailer’s side, introducing the factor of supplier risk aversion behavior, and respectively constructing and solving the Stackelberg game models under wholesale price contract, cost-sharing contract, revenue-sharing contract, and transfer payment contract to explore how government subsidies and risk aversion affect the optimal decisions of members, the freshness of products, and the utility level of the supply chain. The research results indicate that: (1) The increase in government subsidies has a positive impact on the operation of the fresh product supply chain, which will raise wholesale prices, freshness preservation efforts, selling prices, and the overall utility level of the supply chain. (2) The risk aversion of supplier leads to a reduction in transfer payment costs, wholesale prices, and freshness preservation efforts, but this will, in turn, prompt retailer to lower selling prices, thereby enhancing the utility levels of both the retailer and the supply chain entity. (3) The freshness preservation effort by the supplier is higher in transfer payment contracts and cost-sharing contracts with a high cost-sharing ratio. Under transfer payment contracts, supplier and supply chain entity achieve the highest utility levels, while the most efficient utility level of retailer depends on the risk aversion threshold of supplier. When this threshold is exceeded, it will switch to cost-sharing contracts.

## 1. Introduction

In recent years, the significant problem of huge loss in quantity and quality of fresh products has garnered considerable attention [1,2]. Research indicates that establishing cold storage and preservation facilities at the point of origin can effectively mitigate these losses [3]. However, the substantial investment required for cold storage and cold chain infrastructure, coupled with lengthy payback periods and high operational costs, poses challenges that farmers and other suppliers often cannot surmount independently. This is particularly critical given that many farmers and suppliers are risk-averse [4], meaning they are reluctant to invest in high-cost infrastructure due to uncertainties in market demand, price fluctuations, and potential financial losses. Such risk aversion further exacerbates the problem of fresh product loss and limits the adoption of advanced preservation technologies.

To alleviate these financial burdens and address the risk aversion of suppliers, the Chinese government has implemented various subsidies. Notably, the 2020 Implementation Opinions on Accelerating the Construction of Warehousing and Freshness Cold Chain Facilities for Agricultural Products, issued by the Ministry of Agriculture and Rural Affairs, seeks to enhance the development of such facilities for perishable goods like fruits and vegetables across multiple regions, offering financial support of up to 30% of the total investment. These subsidies aim to reduce the financial risks faced by farmers and other fresh product suppliers, encouraging them to invest in cold chain infrastructure. However, the effectiveness of these subsidies in mitigating risk aversion and promoting sustainable investment remains a critical area of study.

Additionally, in the context of social capital, retailers enhance support through contractual mechanisms such as “cost-sharing,” “benefit-sharing,” and “transfer payments.” For instance, Walmart collaborates with suppliers to jointly cover a portion of preservation costs, thereby ensuring the freshness and quality of perishable product. Similarly, Freshippo engages with suppliers to establish sales targets, sharing revenues based on sales performance. Furthermore, Yonghui Supermarket provides upfront payments to suppliers of fresh agricultural products to facilitate the upgrading of agricultural facilities, enhance the quality of fresh goods, and implement advanced preservation technologies and equipment. These contractual mechanisms not only distribute financial risks but also align the incentives of suppliers and retailers, fostering collaboration and innovation in the supply chain.

Consequently, investigating the impacts of government subsidies and various trading contractual mechanisms on fresh product supply chains has emerged as a pressing practical issue. This exploration entails examining how risk-averse suppliers determine pricing and allocate investments toward freshness efforts in light of government subsidy policies and differing contracts. It also involves understanding how retailers adapt their marketing strategies in response to these dynamics and identifying which contract is the most effective in incentivizing suppliers to enhance their preservation initiatives. In the fresh product supply chain, due to the perishability and time-limited nature of the products, the decisions made by the members of the supply chain often have a sequence and mutual influence among each other. By applying the Stackelberg model, this decision-making process can be simulated, and the impact of different decisions and multiple factors on the efficiency of the supply chain can be explored.

Addressing above questions will not only contribute to optimizing fresh product supply chain management and promote sustainable development within China’s current subsidy policy and social context, but also offer valuable insights and lessons for the international community in reducing fresh product losses and improving overall food quality. Moreover, the broader context of supply chain resilience and sustainability highlights the urgency of this research [5]. Global food systems are increasingly vulnerable to disruptions caused by climate change [6], economic instability [7], and geopolitical tensions. Enhancing the resilience of fresh product supply chains—ensuring they can withstand and recover from shocks—is essential for food security and economic stability. Simultaneously, sustainability concerns, such as reducing food waste and minimizing the environmental impact of agricultural practices, are critical for achieving long-term global development goals. By integrating risk management, government subsidies, and contractual mechanisms, this research aims to build more resilient and sustainable supply chains that can adapt to future challenges while minimizing fresh product losses and maximizing resource efficiency.

## 2. Literature review

### 2.1. Government subsidies

Government subsidies have garnered significant attention as a crucial factor influencing the decision-making processes of fresh product supply chain participants. Research indicates that appropriate government subsidies can not only enhance crop yields [8], but also effectively boost farmers’ efforts and increase the profitability of supply chain members [9]. Specifically, increased government support for initial investments in freshness at the origin is viewed as particularly beneficial in minimizing losses [10]. To achieve success, members of the supply chain cannot act independently. Instead, they must work together in a coordinated manner to gain a competitive edge [5]. Given the differing return objectives among supply chain members, many scholars have sought to design contracts that facilitate coordination in the context of government subsidies. For instance, Wu [11] and Yang [12] examined the impact of revenue-sharing contracts on supply chain dynamics under government subsidy policies, while Nie et al. [13] analyzed the effectiveness of wholesale price contracts in coordinating the agricultural supply chain within such frameworks. Additionally, Zhang et al. [14] proposed a “cost-sharing” and “benefit-sharing + transfer payments” contract to enhance subsidy effectiveness, and Xiong et al. [15] investigated how government subsidies for cold chain facilities influence the stability of relational contracts across various organizational models.

However, many of the aforementioned studies operated under the assumption of supplier risk neutrality, neglecting critical factors such as demand risk [16] and the influence of climate on product quality and quantity [17]. In reality, risk-averse behavior is common among upstream supply chain participants, including farmers and cooperatives. Consequently, a deeper exploration of suppliers’ risk aversion characteristics is essential for gaining a more comprehensive understanding of how government subsidy policies impact fresh product supply chain management and their practical implications. By incorporating these risk considerations, researchers can better elucidate the mechanisms through which subsidies affect supply chain dynamics and ultimately enhance decision-making processes among stakeholders.

### 2.2. Risk-averse behaviors

Supply chains are often affected by unpredictable events, which can adversely impact their ability to achieve performance targets [7]. In the fresh product supply chain, the costs associated with cold chain transportation and the construction of cold storage facilities for fresh product are currently substantial. Such investments not only elevate operational expenditures but also introduce market risks and uncertainties [18]. As primary producers of fresh product, farmers and cooperatives often exhibit risk-averse behaviors in response to these uncertainties [19–21]. For instance, Ye et al. [22,23] demonstrated that farmers with a risk-averse inclination tend to increase their optimal yield in response to rising order prices. Conversely, Dan et al. [24] indicated that this risk-averse disposition among producers could exacerbate the double marginalization effect within the supply chain, ultimately diminishing overall profitability. Furthermore, Chen et al. [25] illustrated those heightened levels of farmer risk aversion, coupled with diminished consumer preferences, may hinder the diffusion of innovations in eco-agricultural technologies.

However, suppliers’ risk-averse behavior is not a complete hindrance to the development of fresh product supply chain. For instance, Bai et al. [19] demonstrated that farmers’ risk aversion is inversely related to the level of green investment in decentralized supply chains, yet positively correlated in two types of cost-sharing contracts. Furthermore, Liao et al. [4] noted that the potential for supply chains to achieve Pareto improvements expands as the coefficient of farmers’ risk aversion increases. Consequently, it becomes imperative to delve into the effects of government subsidies and various trading contract mechanisms on decision-making in fresh product supply chain management, particularly in light of supplier’s risk aversion.

### 2.3. Summary

In summary, while government subsidies have garnered considerable academic interest in the context of transaction contract design within fresh product supply chains, a significant research gap persists in the exploration of suppliers’ risk-averse behavior. Despite the recognized importance of considering risk aversion in understanding supply chain dynamics and policy impacts, the majority of existing studies continue to operate under the assumption of supplier risk neutrality. This oversight is problematic because risk-averse behavior is a prevalent trait among upstream supply chain participants, such as farmers and cooperatives, who are often directly impacted by government subsidy policies. By neglecting critical factors like demand risk and the influence of climate on product quality and quantity, these studies fail to capture the full spectrum of challenges and opportunities presented by risk aversion in fresh product supply chains. Moreover, the interplay between risk aversion, government subsidies, and various trading contract mechanisms remains understudied, despite its potential to significantly influence decision-making processes and supply chain efficiency.

To addresses this gap, it is necessary to conduct a more in-depth exploration of the risk-averse characteristics of suppliers. By incorporating risk factors into the research model, scholars can more comprehensively understand how government subsidy policies and contractual mechanisms affect the management of the fresh product supply chain, and ultimately enhance the efficiency of the decision-making process of stakeholders. Therefore, based on the background of government subsidies for fresh product suppliers for product preservation, considering four types of contracts provided by retailers (namely, wholesale price contract, cost-sharing contract, revenue-sharing contract, and transfer payment contract), this paper introduces the risk-averse elements of suppliers, respectively constructs and solves Stackelberg game models under different contracts, and analyzes how risk-averse suppliers determine pricing and preservation investment strategies, as well as how retailers formulate corresponding sales strategies.

This study diverges from previous research in two significant ways. First, existing literature on government subsidies and transaction contract design rarely addresses the risk aversion behaviors of fresh product suppliers. By examining the pricing and preservation input decisions of these risk-averse suppliers, this paper aligns the research more closely with real-world scenarios. Second, in contrast to the commonly analyzed revenue-sharing and cost-sharing contracts, this paper introduces a transfer payment contract for comparative analysis. The findings suggest that the transfer payment contract is more effective in incentivizing suppliers to enhance their freshness efforts, thereby offering a novel perspective on transaction contract design within fresh supply chains and contributing to the advancement of research in this domain.

The paper is structured as follows: Section 3 delineates the research questions and assumptions and is accompanied by the notation. In Section 4, we develop and solve mathematical models under four distinct transaction contracts: wholesale price, cost-sharing, revenue-sharing, and transfer payment. Subsequently, Section 5 delves into analyzing and validating of the impacts of various transaction contracts on supplier’s utility levels. Furthermore, it explores the effects of government subsidies, risk aversion, and contract parameters on optimal decisions and utility levels within fresh product supply chains, focusing mainly on scenarios involving cost-sharing and revenue-sharing contracts. Section 6 presents conclusions, recommendations, and potential avenues for future research. Proofs of all results are furnished in the Appendix.

## 3. Problem description and notation

### 3.1. Problem description and assumptions

**Fig 1:**
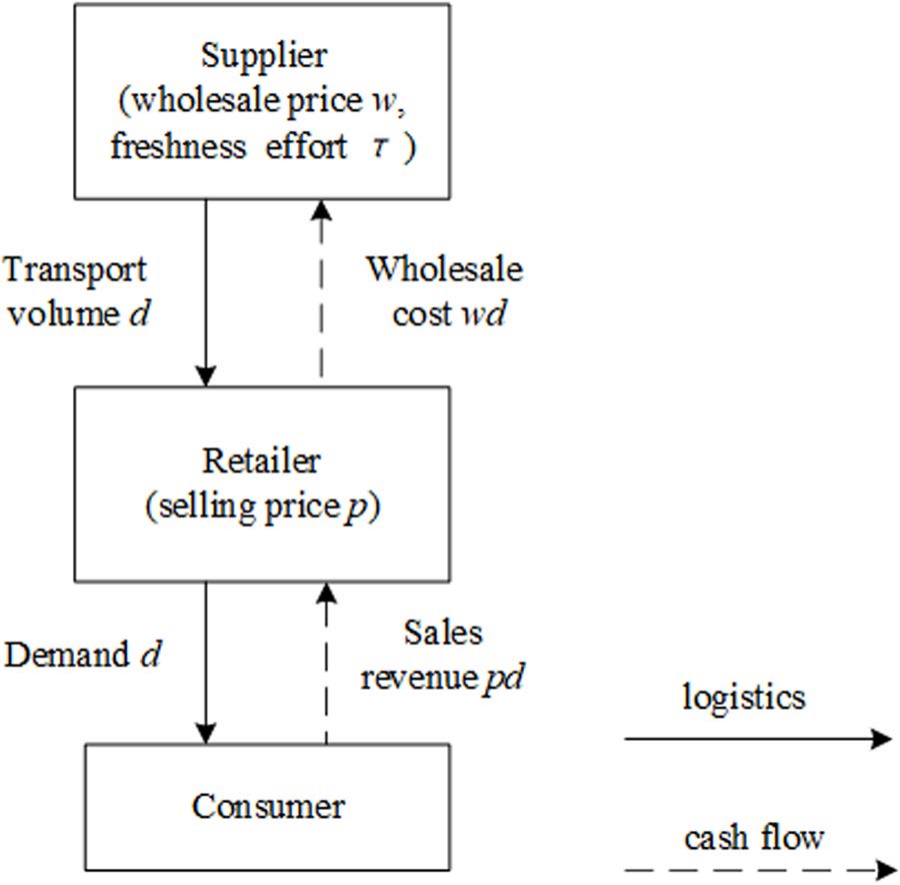
Two-tier fresh product supply chain mode.

Consider a two-tier fresh product supply chain consisting of a fresh product supplier (hereinafter referred to as “supplier” and denoted by “S”) and a fresh product retailer (hereinafter referred to as “retailer” and denoted by “R”) as shown in Fig 1. The supplier is responsible for supplying and transporting fresh products, and the retailer orders fresh products from the supplier and sell them to consumers. During the process of fresh products from “field” to “table”, the supplier undertakes all the freshness handling of fresh products, which is manifested in maintaining the freshness of the products by handling clean vegetables, processing, packaging, pre-cooling and refrigerated storage.

This paper is based on the following hypotheses:

**Hypothesis 1:** Given the government’s role as a proponent and supporter of enhancing the freshness preservation capabilities at the source, various subsidy mechanisms are employed to incentivize supplier to establish cold storage and preservation facilities, and introduce pre-cooling and preservation technologies. The overarching goal is to mitigate losses incurred during the circulation of fresh products. Consequently, it is assumed that the primary target of government subsidies is the supplier. Building upon the findings of Zhang et al. [14], it is further assumed that the government determines and publicizes the subsidy ratio z based on fiscal considerations and market expectations. Subsequently, the government refrains from intervening in the decision-making processes of supply chain members throughout the production and marketing phases of fresh agricultural products.

**Hypothesis 2:** The Supplier is assumed to be risk-averse and the retailer is risk-neutral. References [26–28] use the mean-variance method to measure utility $U(\pi_{i})$. Specifically: $U(\pi_{i})=E(\pi_{i})-\lambda Var(\pi_{i}),(i=S,R)$. Where, $E(\pi_{i})$ represents the expected profit. λ(λ≥0) serves as a measure of risk aversion. λ=0 indicates risk-neutrality, while larger values of λ correspond to higher degrees of risk aversion. $Var(\pi_{i})$ denotes the profit variance, representing the risk loss of the decision-maker. The larger the variance, the greater the volatility of profits, and thus the higher the risk. Conversely, the smaller the variance, the more stable the profits are, and the lower the risk. The calculation formula is $Var(\pi_{i})=E[{\left(\pi_{i}-E(\pi_{i})\right)}^{2}]$.

**Hypothesis 3:** Building upon the insights from Dong et al. [29], the freshness decay function of fresh products under the supplier’s freshness effort is formulated as $\theta (\tau ,t)=\theta_{0}+\eta \tau -\varphi_{0}{\left(\frac{t}{T}\right)}^{2},t\in [0,T]$. Where, $\theta_{0}$ represents the initial freshness value of fresh product when not preserved, τ denotes the level of supplier freshness effort, as evidenced by the supplier’s maintenance of freshness through storage methods such as netting, processing, packaging, and pre-cooling and refrigeration. η represents the factor influencing supplier freshness effort on freshness of the fresh product. $\varphi_{0}$ represents the extreme value of freshness decay after a fixed sales cycle T, and the larger the value, the lower the freshness of the product at the end of the sales cycle.

**Hypothesis 4:** In alignment with the definitions provided by Chambers, He, and Yu [9,30,31], the cost of freshness is modeled as a quadratic function of the freshness effort, which satisfies $c^{\prime }(\tau )>0,c^{\prime \prime }(\tau )>0$, expressed as $c(\tau )=(k\tau^{2})/2$. Where, k(k>0) denotes the freshness effort cost factor, reflecting the relationship between freshness effort and associated costs.

**Hypothesis 5:** Drawing insights from the research conducted by Zheng and Qu et al. [32,33], the market demand for fresh product is conceptualized as a function of the sales price, freshness of the products, and stochastic factors, formulated as d=a−bp+lθ+ε. Where, a denotes the base demand in the market. b,l represent the price and freshness sensitivity coefficients, respectively. $\varepsilon \sim N(0,\delta^{2})$ accounts for the demand perturbation factors, assumed to follow a normal distribution, indicating that market demand is influenced by price and freshness, with inherent volatility.

**Hypothesis 6:** The values of the relevant parameters meet the primary conditions for an optimal solution to the decision.

### 3.2. Description of symbols

The main symbols involved in this paper are described in Table 1:

**Table 1:** Symbol description.

Parameters	Descriptions
p	Unit sales price of fresh product.
w	Unit wholesale price of fresh product.
τ	Freshness preservation effort.
γ	Cost-sharing ratio.
χ	Revenue-sharing ratio.
F	Transfer payment fee
λ	Degree of risk aversion.
z	Government subsidy coefficient, 0<z<1.
t	0<t≤T The moment when a fresh product is in the middle of the sales cycle T.
T	Fresh product lifecycle.
$\theta_{0}$	Initial freshness of fresh product
$\varphi_{0}$	Fresh product freshness decay extremes.
η	Parameters of the effect of freshness effort on rawness.
k	Freshness effort cost factor.
a	Market-based demand.
b	Consumer price sensitivity factor.
l	Freshness sensitivity factor
ε	Demand stochastic perturbation factor
$\pi_{i}$	Revenue function

The superscript “*” indicates the optimal value. j=(PF,CB,SY,PZ) denote wholesale price contract, cost-sharing contract, benefit-sharing contract, and transfer payment contract, respectively. Subscript i=(S,R,O) denote supplier, retailer, and the whole fresh product supply chain, respectively.

## 4. Model construction and analysis

In this paper, we discuss the optimal decisions of the supplier and the retailer separately when the supplier provides fresh product to the retailer only at wholesale price and there are no other transactions between the two (Scenario 1, denoted by superscript “PF”), the retailer shares part of the supplier’s freshness preservation cost of fresh products (Scenario 2, denoted by the superscript “CB”), when the retailer shares part of the sales revenue to the supplier (Scenario 3, superscript “SY”), and when the retailer pays a certain amount of fees in advance to the supplier (Scenario 4, superscript “PZ”).

**Fig 2:**
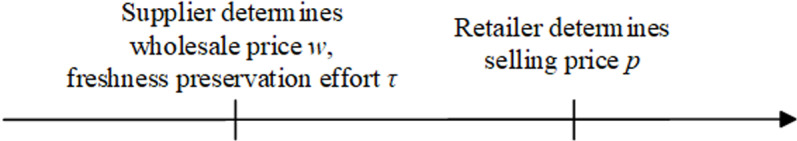
Decision sequence for supplier-retailer two-tier fresh product supply chain.

This paper constructs a Stackelberg game model between a supplier and a retailer, assuming that the supplier is the game leader and the retailer is the game follower. The decision-making sequence is that the supplier, aiming to maximize utility, simultaneously decides on the wholesale price of the fresh products and its level of preservation effort, and then the retailer decides on the final selling price based on the supplier’s decision-making information (Fig 2).

### 4.1. Wholesale price contract model (PF model)

One of the most widely used forms of transaction in current business practice is the wholesale price contract, namely that, the supplier provides the retailer with the fresh products at wholesale prices based on the number of orders placed by the retailer, and the retailer sells the products to the final consumer after adding a certain profit margin to the wholesale price. Under the wholesale price contract, the utility functions of the supplier and the retailer are, respectively.


$$U(\pi_{S}^{PF})=w(a-bp+l(\theta_{0}+\eta \tau -\varphi_{0}{\left(\frac{t}{T}\right)}^{2}))-(1-z)\frac{k\tau^{2}}{2}-\lambda w^{2}\delta^{2}$$ (1)

$$U(\pi_{R}^{PF})=(p-w)(a-bp+l(\theta_{0}+\eta \tau -\varphi_{0}{\left(\frac{t}{T}\right)}^{2}))$$ (2)

In the following, the above model is solved by backward induction, and the equilibrium result is shown in Theorem 1.

**Theorem 1** In the wholesale price contract,


$$w^{PF*}=\frac{2k(-1+z)(T^{2}(a+l\theta_{0})-lt^{2}\varphi_{0})}{T^{2}(l^{2}\eta^{2}+4k(-1+z)(b+2\lambda \delta^{2})}$$

$$\tau^{PF*}=\frac{l\eta (-T^{2}(a+l\theta_{0})+lt^{2}\varphi_{0})}{T^{2}(l^{2}\eta^{2}+4k(-1+z)(b+2\lambda \delta^{2})}$$

$$p^{PF*}=\frac{k(-1+z)(3b+4\lambda \delta^{2})(T^{2}(a+l\theta_{0})-lt^{2}\varphi_{0})}{bT^{2}(4bk(-1+z)+l^{2}\eta^{2}+8k(-1+z)\lambda \delta^{2})}$$

$$\theta^{PF*}=\theta_{0}-\varphi_{0}\frac{t^{2}}{T^{2}}+\frac{l\eta^{2}(-T^{2}(a+l\theta_{0})+lt^{2}\varphi_{0})}{T^{2}(l^{2}\eta^{2}+4k(-1+z)(b+2\lambda \delta^{2}))}$$

$$U(\pi_{S}^{PF*})=\frac{k(-1+z){\left(T^{2}(a+l\theta_{0})-lt^{2}\varphi_{0}\right)}^{2}}{2T^{4}(4bk(-1+z)+l^{2}\eta^{2}+8k(-1+z)\lambda \delta^{2})}$$

$$U(\pi_{R}^{PF*})=\frac{k^{2}{\left(-1+z\right)}^{2}{\left(b+4\lambda \delta^{2}\right)}^{2}{\left(T^{2}(a+l\theta_{0})-lt^{2}\varphi_{0}\right)}^{2}}{bT^{4}{\left(4bk(-1+z)+l^{2}\eta^{2}+8k(-1+z)\lambda \delta^{2}\right)}^{2}}$$

$$\begin{array}{cc} & U(\pi_{O}^{PF*})=(k(-1+z)(6b^{2}k(-1+z)+bl^{2}\eta^{2}+24bk(-1+z)\delta^{2}\lambda +32k(-1+z)\delta^{4}\lambda^{2})\\ & \;\;\;{\left(T^{2}(a+l\theta_{0})-lt^{2}\varphi_{0}\right)}^{2})/(2bT^{4}{\left(l^{2}\eta^{2}+4k(-1+z)(b+2\lambda \delta^{2})\right)}^{2}) \end{array}$$

All proofs can be found in the Appendix.

Taking derivative with respect to the risk aversion coefficient λ on $w^{PF*},\tau^{PF*},p^{PF*},\theta^{PF*},U(\pi_{S}^{PF*}),U(\pi_{R}^{PF*}),U(\pi_{O}^{PF*})$, we can obtain Corollary 1.

Corollary 1 $\frac{\partial w^{PF*}}{\partial \lambda }<0$, $\frac{\partial \tau^{PF*}}{\partial \lambda }<0$, $\frac{\partial p^{PF*}}{\partial \lambda }<0$, $\frac{\partial \theta^{PF*}}{\partial \lambda }<0$, $\frac{\partial U(\pi_{S}^{PF*})}{\partial \lambda }<0$. When $0<k<\frac{l^{2}\eta^{2}}{2b(1-z)}$, $\frac{\partial U(\pi_{R}^{PF*})}{\partial \lambda }<0$, $\frac{\partial U(\pi_{O}^{PF*})}{\partial \lambda }<0$; when $\frac{l^{2}\eta^{2}}{2b(1-z)}<k<-\frac{l^{2}\eta^{2}(b+8\lambda \delta^{2})}{8b(-1+zlambda\delta^{2}}$, $\frac{\partial U(\pi_{R}^{PF*})}{\partial \lambda }>0$, $\frac{\partial U(\pi_{O}^{PF*})}{\partial \lambda }<0$; when $k>-\frac{l^{2}\eta^{2}(b+8\lambda \delta^{2})}{8b(-1+zlambda\delta^{2}}$, $\frac{\partial U(\pi_{R}^{PF*})}{\partial \lambda }>0$, $\frac{\partial U(\pi_{O}^{PF*})}{\partial \lambda }>0$.

According to Corollary 1, we can acknowledge that in the wholesale price contract model, the wholesale price, the freshness preservation effort, the selling price, the freshness of the product, and the supplier’s utility level exhibit negative correlations with the supplier’s risk aversion coefficient, whereas when the cost coefficient of the supplier’s freshness preservation effort exceeds a threshold $-\frac{l^{2}\eta^{2}(b+8\lambda \delta^{2})}{8b(-1+zlambda\delta^{2}}$, the utility level of the retailer and the supply chain entirety exhibit positive correlations with the supplier’s risk aversion coefficient.

When the supplier shows a risk-averse tendency, its decision-making behavior tends to be conservative, on the one hand, avoiding the risk of freshness investment by reducing the freshness preservation effort, and on the other hand, reducing the risk of sales by lowering the wholesale price. As the reduction in freshness effort leads to a decrease in freshness of fresh product, the retailer is forced to adopt a price reduction strategy to stimulate consumer demand in order to maintain sales volume and utility level. As supplier become more risk-averse, this conservative decision-making leads to a steady decline in freshness and wholesale price, and the retailer is required to continually reduce its selling price in response to changes in the marketplace. This price reduction strategy is only conducive to the development of the supply chain under a certain condition, namely that, when the fresh-ability of the supplier is lower than a certain level (i.e., $k>-\frac{l^{2}\eta^{2}(b+8\lambda \delta^{2})}{8b(-1+zlambda\delta^{2}}$), the supplier’s risk aversion can promote the improvement of the utility level of the retailer and the supply chain system. This shows that the conservative decisions taken by the risk-averse supplier to deal with risks will have a positive impact on the retailer and the supply chain entirety under a certain condition.

Taking derivative with respect to the government subsidy coefficient z on $w^{PF*},\tau^{PF*},p^{PF*},\theta^{PF*},U(\pi_{S}^{PF*}),U(\pi_{R}^{PF*}),U(\pi_{O}^{PF*})$, we can obtain Corollary 2.

Corollary 2$\frac{\partial w^{PF*}}{\partial z}>0$, $\frac{\partial \tau^{PF*}}{\partial z}>0$, $\frac{\partial p^{PF*}}{\partial z}>0$, $\frac{\partial \theta^{PF*}}{\partial z}>0$, $\frac{\partial U(\pi_{S}^{PF*})}{\partial z}>0$, $\frac{\partial U(\pi_{R}^{PF*})}{\partial z}>0$, $\frac{\partial U(\pi_{O}^{PF*})}{\partial z}>0$.

As shown in Corollary 2, in the wholesale price contract, the wholesale price, the freshness preservation effort, the selling price, the freshness of the product, as well as the utility levels of the supplier, retailer and the supply chain entirety, all increase with the proportion of government subsidy on preservation costs. This result suggests that government subsidies, supported by the policy of subsidizing cold storage and preservation facilities at the place of origin, provide a significant incentive to supplier’s freshness effort, that is, the greater the government subsidy, the more supplier tends to increase its freshness preservation effort, and the increased freshness effort enhances product freshness. However, the increase in freshness effort also leads to an increase in supplier’s preservation cost, and the supplier passes on this additional cost to the retailer, which is manifested in higher wholesale price. The retailer then passes this cost on to the end consumer, resulting in higher selling price. It can be seen that government subsidies not only improve the freshness level of fresh product to achieve effective control of fresh product wastage, but also can effectively enhance the efficiency of the supply chain.

### 4.2. Cost-sharing contract model (CB model)

Under the cost-sharing contract, the retailer bears a certain proportion of the suppliers’ freshness costs, thereby reducing the supplier’s cost burden and improving the overall efficiency of the supply chain. For example, Walmart, one of the world’s leading retailers, has taken the initiative to bear part of the preservation costs by investing in state-of-the-art cold chain logistics systems and storage facilities to ensure the quality and freshness of fresh products. In addition, Walmart works closely with suppliers to develop preservation measures, including appropriate product packaging and storage conditions, to extend the shelf life of products. In this contract, the retailer’s cost-sharing ratio is γ, and the utility functions of the supplier and retailer are, respectively,


$$U(\pi_{S}^{CB})=w(a-bp+l(\theta_{0}+\eta \tau -\varphi_{0}{\left(\frac{t}{T}\right)}^{2}))-(1-z)(1-\gamma )\frac{k\tau^{2}}{2}-\lambda w^{2}\delta^{2}$$ (3)

$$U(\pi_{R}^{CB})=(p-w)(a-bp+l(\theta_{0}+\eta \tau -\varphi_{0}{\left(\frac{t}{T}\right)}^{2}))-\gamma (1-z)\frac{k\tau^{2}}{2}$$ (4)

In the following, the above model is solved by backward induction, and the equilibrium result is shown in Theorem 2.

**Theorem 2** In the cost-sharing contract,


$$w^{CB*}=\frac{2k(-1+z)(-1+\gamma )(T^{2}(a+l\theta_{0})-lt^{2}\varphi_{0})}{T^{2}(-l^{2}\eta^{2}+4k(-1+z)(-1+\gamma )(b+2\lambda \delta^{2}))}$$

$$\tau^{CB*}=\frac{l\eta (T^{2}(a+l\theta_{0})-lt^{2}\varphi_{0})}{T^{2}(-l^{2}\eta^{2}+4k(-1+z)(-1+\gamma )(b+2\lambda \delta^{2}))}$$

$$p^{CB*}=\frac{k(-1+z)(-1+\gamma )(3b+4\lambda \delta^{2})(T^{2}(a+l\theta_{0})-lt^{2}\varphi_{0})}{bT^{2}(4bk(-1+z)(-1+\gamma )-l^{2}\eta^{2}+8k(-1+z)(-1+\gamma )\lambda \delta^{2})}$$

$$\theta^{CB*}=\theta_{0}-\varphi_{0}\frac{t^{2}}{T^{2}}+\frac{l\eta^{2}(T^{2}(a+l\theta_{0})-lt^{2}\varphi_{0})}{T^{2}(-l^{2}\eta^{2}+4k(-1+z)(-1+\gamma )(b+2\lambda \delta^{2}))}$$

$$U(\pi_{S}^{CB*})=\frac{k(-1+z)(-1+\gamma ){\left(T^{2}(a+l\theta_{0})-lt^{2}\varphi_{0}\right)}^{2}}{2T^{4}(4bk(-1+z)(-1+\gamma )-l^{2}\eta^{2}+8k(-1+z)(-1+\gamma )\lambda \delta^{2})}$$

$$\begin{array}{cc} & U(\pi_{R}^{CB*})=(k(-1+z)(2b^{2}k(-1+z){\left(-1+\gamma \right)}^{2}+bl^{2}\eta^{2}\gamma +16k(-1+z){\left(-1+\gamma \right)}^{2}\lambda \delta^{2}+32k\\ & \;\;\;\;(-1+z){\left(-1+\gamma \right)}^{2}\lambda^{2}\delta^{4}){\left(T^{2}(a+l\theta_{0})-lt^{2}\varphi_{0}\right)}^{2})/(2bT^{4}(4bk(-1+z)(-1+\gamma )-l^{2}\eta^{2}+\\ & \;\;\;\;8k(-1+z)(-1+\gamma )\lambda \delta^{2}{)}^{2}) \end{array}$$

$$\begin{array}{cc} & U(\pi_{O}^{CB*})=(k(-1+z)(6b^{2}k(-1+z){\left(-1+\gamma \right)}^{2}+bl^{2}\eta^{2}\gamma +24bk(-1+z){\left(-1+\gamma \right)}^{2}\lambda \delta^{2}+32k\\ & \;\;\;\;(-1+z){\left(-1+\gamma \right)}^{2}\lambda^{2}\delta^{4}){\left(T^{2}(a+l\theta_{0})-lt^{2}\varphi_{0}\right)}^{2})/(2bT^{4}(4bk(-1+z)(-1+\gamma )-l^{2}\eta^{2}+\\ & \;\;\;\;8k(-1+z)(-1+\gamma )\lambda \delta^{2}{)}^{2}) \end{array}$$

$$w^{CB*}=\frac{2k(-1+z)(-1+\gamma )(T^{2}(a+l\theta_{0})-lt^{2}\varphi_{0})}{T^{2}(-l^{2}\eta^{2}+4k(-1+z)(-1+\gamma )(b+2\lambda \delta^{2}))}$$

Taking derivative with respect to the risk aversion coefficient λ on $w^{CB*},\tau^{CB*},p^{CB*},\theta^{CB*},U(\pi_{S}^{CB*}),U(\pi_{R}^{CB*}),U(\pi_{O}^{CB*})$, we can obtain Corollary 3.

Corollary 3 $\frac{\partial w^{CB*}}{\partial \lambda }<0$, $\frac{\partial \tau^{CB*}}{\partial \lambda }<0$, $\frac{\partial p^{CB*}}{\partial \lambda }<0$, $\frac{\partial \theta^{CB*}}{\partial \lambda }<0$, $\frac{\partial U(\pi_{S}^{CB*})}{\partial \lambda }<0$

From Corollary 3, it can be observed that, similar to Corollary 1, in the cost-sharing contract, the wholesale price, the freshness preservation effort, the selling price, the freshness of the product, and the supplier’s utility level are inversely proportional to the supplier’s risk aversion coefficient. However, due to the complexity of the mathematical expressions for the derivative of the utility levels of the retailer and the supply chain entirety with respect to the risk aversion coefficient, the relationship is not apparent and it is difficult to clearly identify the interrelationship directly from the derivative expressions. Therefore, the dynamic relationship between these variables will be further explored through simulation analyses subsequently.

Taking derivative with respect to the government subsidy coefficient z on $w^{PF*},\tau^{PF*},p^{PF*},\theta^{PF*},U(\pi_{S}^{PF*}),U(\pi_{R}^{PF*}),U(\pi_{O}^{PF*})$, we can obtain Corollary 4.

Corollary 4 $\frac{\partial w^{CB*}}{\partial z}>0$, $\frac{\partial \tau^{CB*}}{\partial z}>0$, $\frac{\partial p^{CB*}}{\partial z}>0$, $\frac{\partial \theta^{CB*}}{\partial z}>0$, $\frac{\partial U(\pi_{S}^{CB*})}{\partial z}>0$

From Corollary 4, it can be observed that, similar to Corollary 2, in the cost-sharing contract, the wholesale price, the freshness preservation effort, the selling price, the freshness of the product, and the supplier’s utility level are directly proportional to the government subsidy. Similarly, due to the complexity of the mathematical expressions for the derivative of the utility levels of the retailer and the supply chain entirety with respect to the government subsidy coefficient, the dynamic relationship between these variables will be further explored through simulation analyses subsequently.

Taking derivative with respect to the cost-sharing ratio γ on $w^{PF*},\tau^{PF*},p^{PF*},\theta^{PF*},U(\pi_{S}^{PF*}),U(\pi_{R}^{PF*}),U(\pi_{O}^{PF*})$, we can obtain Corollary 5.

Corollary 5 $\frac{\partial w^{CB*}}{\partial \gamma }>0$, $\frac{\partial \tau^{CB*}}{\partial \gamma }>0$, $\frac{\partial p^{CB*}}{\partial \gamma }>0$, $\frac{\partial \theta^{CB*}}{\partial \gamma }>0$, $\frac{\partial U(\pi_{S}^{CB*})}{\partial \gamma }>0$

From Corollary 5, it can be observed that, in the cost-sharing contract, the wholesale price, the freshness preservation effort, the selling price, the freshness of the product, and the supplier’s utility level are directly proportional to the cost-sharing ratio. This is because the retailer has taken on part of the preservation costs of the supplier, alleviating the pressure on the supplier in the preservation process, thereby stimulating its enthusiasm for preservation and increasing efforts in preservation. Eventually, this leads to an improvement in the freshness of fresh products and enhances consumers’ purchasing intentions. As the preservation costs increase, both the supplier and the retailer have adopted price increase strategies accordingly. Similarly, due to the complexity of the mathematical expressions for the derivative of the utility levels of the retailer and the supply chain entirety with respect to the cost-sharing ratio, the dynamic relationship between these variables will be further explored through simulation analyses subsequently.

### 4.3. Revenue sharing contract model (SY Model)

Under the revenue sharing contract, the retailer transfers part of the sales revenue to the supplier to incentivize the supplier to improve the freshness of the fresh products. For example, Boxmart Fresh and its suppliers establish a close partnership, jointly set sales targets, and share proceeds in an agreed ratio based on sales performance. This contractual arrangement not only promotes synergy among supply chain members, but also enhances the overall supply chain performance. In this contract, the revenue sharing ratio of the retailer is χ, and the utility functions of the supplier and retailer are, respectively,


$$U(\pi_{S}^{SY})=(w+\chi p)(a-bp+l(\theta_{0}+\eta \tau -\varphi_{0}{\left(\frac{t}{T}\right)}^{2}))-(1-z)\frac{k\tau^{2}}{2}-\lambda {\left(w+\chi p\right)}^{2}\delta^{2}$$ (5)

$$U(\pi_{R}^{SY})=((1-\chi )p-w)(a-bp+l(\theta_{0}+\eta \tau -\varphi_{0}{\left(\frac{t}{T}\right)}^{2}))$$ (6)

In the following, the above model is solved by backward induction, and the equilibrium result is shown in Theorem 3.

**Theorem 3** Under the revenue sharing contract,


$$w^{SY*}=\frac{2k(-1+z)(-1+\chi )(b-b\chi +\lambda \delta^{2}(-2+\chi )\chi )(T^{2}(a+l\theta_{0})-lt^{2}\varphi_{0})}{bT^{2}(-l^{2}\eta^{2}+2k(-1+z)(b-\lambda \delta^{2}(-2+\chi ))(-2+\chi ))}$$

$$\tau^{SY*}=\frac{l\eta (T^{2}(a+l\theta_{0})-lt^{2}\varphi_{0})}{T^{2}(-l^{2}\eta^{2}+2k(-1+z)(b-\lambda \delta^{2}(-2+\chi ))(-2+\chi ))}$$

$$p^{SY*}=\frac{k(-1+z)(b(3-2\chi )+2\lambda \delta^{2}(2-3\chi +\chi^{2}))(T^{2}(a+l\theta_{0})-lt^{2}\varphi_{0})}{bT^{2}(l^{2}\eta^{2}+2k(-1+z)(-b+\lambda \delta^{2}(-2+\chi ))(-2+\chi ))}$$

$$\theta^{SY*}=\theta_{0}-\varphi_{0}\frac{t^{2}}{T^{2}}+\frac{l\eta^{2}(T^{2}(a+l\theta_{0})-lt^{2}\varphi_{0})}{T^{2}(-l^{2}\eta^{2}+2k(-1+z)(b-\lambda \delta^{2}(-2+\chi ))(-2+\chi ))}$$

$$U(\pi_{S}^{SY*})=-\frac{k(-1+z){\left(T^{2}(a+l\theta_{0})-lt^{2}\varphi_{0}\right)}^{2}}{2T^{4}(-l^{2}\eta^{2}+2bk(-1+z)(-2+\chi )-2k(-1+z)\lambda \delta^{2}{\left(-2+\chi \right)}^{2})}$$

$$U(\pi_{R}^{SY*})=-\frac{k^{2}{\left(-1+z\right)}^{2}{\left(b-2\lambda \delta^{2}(-2+\chi )\right)}^{2}(-1+\chi ){\left(T^{2}(a+l\theta_{0})-lt^{2}\varphi_{0}\right)}^{2}}{bT^{4}{\left(l^{2}\eta^{2}-2bk(-1+z)(-2+\chi )+2k(-1+z)\lambda \delta^{2}{\left(-2+\chi \right)}^{2}\right)}^{2}}$$

$$\begin{array}{cc} & U(\pi_{O}^{SY*})=-((k(-1+z)(8k(-1+z)\delta^{4}\lambda^{2}{\left(-2+\chi \right)}^{2}(-1+\chi )+2b^{2}k(-1+z)(-3+2\chi )-b\\ & \;\;\;(l^{2}\eta^{2}+2k(-1+z)\lambda \delta^{2}(12-16\chi +5\chi^{2}))){\left(T^{2}(a+l\theta_{0})-lt^{2}\varphi_{0}\right)}^{2})/(2bT^{4}(l^{2}\eta^{2}-2bk\\ & \;\;\;(-1+z)(-2+\chi )+2k(-1+z)\lambda \delta^{2}{\left(-2+\chi \right)}^{2}{)}^{2} \end{array}$$

Taking derivative with respect to the risk aversion coefficient λ on $w^{SY*},\tau^{SY*},p^{SY*},\theta^{SY*},U(\pi_{S}^{SY*}),U(\pi_{R}^{SY*}),U(\pi_{O}^{SY*})$, we can obtain Corollary 6.

Corollary 6 $\frac{\partial \tau^{SY*}}{\partial \lambda }<0$, $\frac{\partial p^{SY*}}{\partial \lambda }<0$, $\frac{\partial \theta^{SY*}}{\partial \lambda }<0$, $\frac{\partial U(\pi_{S}^{SY*})}{\partial \lambda }<0$. When $0<k<\frac{l^{2}\eta^{2}\chi }{2b(-1+z)(-2+\chi )}$, $\frac{\partial w^{SY*}}{\partial \lambda }>0$, $\frac{\partial U(\pi_{R}^{SY*})}{\partial \lambda }<0$; when $\frac{l^{2}\eta^{2}\chi }{2b(-1+z)(-2+\chi )}<k<\frac{l^{2}\eta^{2}}{b(-1+z)(-2+\chi )}$, $\frac{\partial w^{SY*}}{\partial \lambda }<0$, $\frac{\partial U(\pi_{R}^{SY*})}{\partial \lambda }<0$; when $k>\frac{l^{2}\eta^{2}}{b(-1+z)(-2+\chi )}$, $\frac{\partial w^{SY*}}{\partial \lambda }<0$, $\frac{\partial U(\pi_{R}^{SY*})}{\partial \lambda }>0$.

According to Corollary 6, we can discover that, similar to Corollary 1, in the revenue-sharing contract model, the freshness preservation effort, the selling price, the freshness of the product, and the supplier’s utility level exhibit negative correlations with the supplier’s risk aversion coefficient, whereas when the cost coefficient of the supplier’s freshness preservation effort exceeds a threshold $\frac{l^{2}\eta^{2}}{b(-1+z)(-2+\chi )}$, the wholesale price is negatively correlated with the risk aversion coefficient, while the utility level of the retailer is positively correlated with the risk aversion coefficient. It can be concluded from this that in a revenue-sharing contract, the supplier’s risk aversion will also lead to a decrease in its efforts to preserve freshness, product freshness, selling price, and its own utility level. Only when its preservation ability is lower than a certain level (i.e., $k>\frac{l^{2}\eta^{2}}{b(-1+z)(-2+\chi )}$), will it have a positive impact on the retailer. Similarly, due to the complexity of the mathematical expression for the derivative of the utility level of the supply chain entirety with respect to the risk aversion coefficient, the dynamic relationship between these variables will be further explored through simulation analyses subsequently.

Taking derivative with respect to the government subsidy coefficient z on $w^{SY*},\tau^{SY*},p^{SY*},\theta^{SY*},U(\pi_{S}^{SY*}),U(\pi_{R}^{SY*}),U(\pi_{O}^{SY*})$, we can obtain Corollary 7.

Corollary 7 $\frac{\partial w^{SY*}}{\partial z}>0$, $\frac{\partial \tau^{SY*}}{\partial z}>0$, $\frac{\partial p^{SY*}}{\partial z}>0$, $\frac{\partial \theta^{SY*}}{\partial z}>0$, $\frac{\partial U(\pi_{S}^{SY*})}{\partial z}>0$, $\frac{\partial U(\pi_{R}^{SY*})}{\partial z}>0$.

From Corollary 7, it can be observed that, similar to Corollary 2 and 4, in the revenue-sharing contract, the wholesale price, the freshness preservation effort, the selling price, the freshness of the product, as well as the utility levels of the supplier and retailer are directly proportional to the government subsidy. Similarly, due to the complexity of the mathematical expression for the derivative of the utility level of the supply chain entirety with respect to the government subsidy coefficient, the dynamic relationship between these variables will be further explored through simulation analyses subsequently.

Taking derivative with respect to the revenue-sharing ratio z on $w^{SY*},\tau^{SY*},p^{SY*},\theta^{SY*},U(\pi_{S}^{SY*}),U(\pi_{R}^{SY*}),U(\pi_{O}^{SY*})$, we can obtain Corollary 8.

Corollary 8 $\frac{\partial \tau^{SY*}}{\partial \chi }>0$, $\frac{\partial \theta^{SY*}}{\partial \chi }>0$, $\frac{\partial U(\pi_{S}^{SY*})}{\partial \chi }>0$.

From Corollary 8, similar to the Corollary 5, in the revenue-sharing contract, the freshness preservation effort, the freshness of the product, and the supplier’s utility all increase with the revenue-sharing ratio. This is because, under a revenue-sharing contract, the higher the retailer’s share of revenue sharing, the larger the share of revenue available to the supplier, which enhances the supplier’s incentive to increase freshness effort. Similarly, due to the complexity of the mathematical expressions for the derivative of the wholesale price, the selling price, as well as the utility levels of the retailer and the supply chain entirety with respect to the revenue-sharing ratio, the dynamic relationship between these variables will be further explored through simulation analyses subsequently.

### 4.4. Transfer payment contract model (PZ model)

In the transfer payment contract, the retailer pays the supplier a certain amount in advance in exchange for the supplier supplying the retailer with products at a lower wholesale price. This contract not only incentivizes suppliers to improve their preservation efforts and lower wholesale prices, but also helps to share the retailer’s risk due to the uncertainty of sales revenue. For example, Yonghui Supermarket often adopts an advance payment to support its fresh produce suppliers, where the funds are used for the improvement of agricultural facilities, optimization of product cultivation, and introduction and upgrading of preservation technology and equipment, and in return, the suppliers provide Yonghui Supermarket with high-quality fresh produce at preferential price. In this contract, the supplier maximizes its utility by making decisions on the amount of transfer payments provided by the retailer, the level of preservation effort, and the wholesale price. Subsequently, the retailer determines the final selling price of the fresh product subject to the satisfaction of the Individual Rationality (IR) and Incentive Compatibility (IC) constraints. The participation constraint ensures that the retailer’s net benefit after paying the transfer fee is not lower than it would have been if the transfer fee had not been paid, while the incentive compatibility constraint ensures that the retailer is able to choose the optimal selling price in order to maximize its own benefit. That is, the above description can be expressed as:


$$\underset{F,w,\tau }{\max}\;U(\pi_{S}^{PZ})=w(a-bp+l(\theta_{0}+\eta \tau -\varphi_{0}{\left(\frac{t}{T}\right)}^{2}))-(1-z)\frac{k\tau^{2}}{2}-\lambda w^{2}\delta^{2}+F$$ (7)

s.t. $\left(IR)U(\pi_{R}^{PZ}geU(\pi_{R}^{PF*}\right)$


$$(IC)\underset{p}{\max}\;U(\pi_{R}^{PZ})=(p-w)(a-bp+l(\theta_{0}+\eta \tau -\varphi_{0}{\left(\frac{t}{T}\right)}^{2}))-F$$ (8)

In the following, the above model is solved by backward induction, and the equilibrium result is shown in Theorem 4.

**Theorem 4** Under the transfer payment contract,


$$w^{PZ*}=0,\;\;\;\tau^{PZ*}=\frac{l\eta (-T^{2}(a+l\theta_{0})+lt^{2}\varphi_{0})}{T^{2}(2bk(-1+z)+l^{2}\eta^{2})}$$

$$p^{SY*}=\frac{k(-1+z)(T^{2}(a+l\theta_{0})-lt^{2}\varphi_{0})}{T^{2}(2bk(-1+z)+l^{2}\eta^{2})}$$

$$F=\frac{k^{2}{\left(-1+z\right)}^{2}}{bT^{4}}(\frac{b^{2}{\left(T^{2}(a+l\theta_{0})-lt^{2}\varphi_{0}\right)}^{2}}{{\left(2bk(-1+z)+l^{2}\eta^{2}\right)}^{2}}-\frac{{\left(b+4\lambda \delta^{2}\right)}^{2}{\left(T^{2}(a+l\theta_{0})-lt^{2}\varphi_{0}\right)}^{2}}{{\left(l^{2}\eta^{2}+4k(-1+z)(b+2\lambda \delta^{2})\right)}^{2}})$$

$$\theta^{PZ*}=\frac{-alT^{2}\eta^{2}+2bkT^{2}(-1+z)\theta_{0})-2bkt^{2}(-1+z)\varphi_{0}}{T^{2}(2bk(-1+z)+l^{2}\eta^{2})}$$

$$\begin{array}{cc} & U(\pi_{S}^{PZ*})=(k(-1+z){\left(T^{2}(a+l\theta_{0})-lt^{2}\varphi_{0}\right)}^{2}(12b^{3}k^{2}{\left(-1+z\right)}^{2}+bl^{4}\eta^{4}-32kl^{2}(-1+z)\delta^{4}\eta^{2}\lambda^{2}+\\ & \;\;\;\;2b^{2}k(1+z)(3l^{2}\eta^{2}+16k(-1+z)\lambda \delta^{2}))/(2bT^{4}(2bk(-1+z)+l^{2}\eta^{2})(4bk(-1+z)+\\ & \;\;\;\;l^{2}\eta^{2}+8k(-1+z)\lambda \delta^{2}{)}^{2}) \end{array}$$

$$U(\pi_{R}^{PZ*})=\frac{k^{2}{\left(-1+z\right)}^{2}{\left(b+4\lambda \delta^{2}\right)}^{2}{\left(T^{2}(a+l\theta_{0})-lt^{2}\varphi_{0}\right)}^{2}}{bT^{4}{\left(4bk(-1+z)+l^{2}\eta^{2}+8k(-1+z)\lambda \delta^{2}\right)}^{2}}$$

$$U(\pi_{O}^{PZ*})=\frac{k(-1+z){\left(T^{2}(a+l\theta_{0})-lt^{2}\varphi_{0}\right)}^{2}}{2T^{4}(2bk(-1+z)+l^{2}\eta^{2})}.$$

Taking derivative with respect to the risk aversion λ on $w^{PZ*},\tau^{PZ*},p^{PZ*},\theta^{PZ*},U(\pi_{S}^{PZ*}),U(\pi_{R}^{PZ*}),U(\pi_{O}^{PZ*})$, we can obtain Corollary 9.

Corollary 9 $\frac{\partial w^{PZ*}}{\partial \lambda }=0$, $\frac{\partial \tau^{PZ*}}{\partial \lambda }=0$, $\frac{\partial p^{PZ*}}{\partial \lambda }=0$, $\frac{\partial \theta^{PZ*}}{\partial \lambda }=0$, $\frac{\partial F}{\partial \lambda }<0$, $\frac{\partial U(\pi_{S}^{PZ*})}{\partial \lambda }<0$, $\frac{\partial U(\pi_{R}^{PZ*})}{\partial \lambda }>0$, $\frac{\partial U(\pi_{O}^{PZ*})}{\partial \lambda }=0$.

As shown in Corollary 9, the supplier’s risk aversion does not directly affect the wholesale price, the freshness preservation effort, the selling price, the freshness of the product, and the utility level of the supply chain entity in the transfer payment contract. However, since the risk mainly arises from the uncertainty of the cost of preservation investment, the risk-averse supplier will still take a more conservative decision, and its required transfer payment charges will decrease with the increase of its risk aversion as a way to reduce the risk of preservation investment. This decision-making pattern leads to a decrease in the supplier’s utility level. In contrast, the retailer’s utility rises as the supplier’s risk aversion increases, as the retailer is able to agree with the supplier at a lower wholesale price, resulting in a higher return. Therefore, in the transfer payment contract, the risk-averse behavior of the supplier is conducive to increasing the utility level of the retailer, but it does not directly affect the overall efficiency of the supply chain.

Taking derivative with respect to the government subsidy coefficient z on $w^{PZ*},\tau^{PZ*},p^{PZ*},\theta^{PZ*},U(\pi_{S}^{PZ*}),U(\pi_{R}^{PZ*}),U(\pi_{O}^{PZ*})$, we can obtain Corollary 10.

Corollary 10 $\frac{\partial w^{PZ*}}{\partial z}=0$, $\frac{\partial \tau^{PZ*}}{\partial z}>0$, $\frac{\partial p^{PZ*}}{\partial z}>0$, $\frac{\partial \theta^{PZ*}}{\partial z}>0$, $\frac{\partial U(\pi_{R}^{PZ*})}{\partial z}>0$, $\frac{\partial U(\pi_{O}^{PZ*})}{\partial z}>0$.

According to Corollary 10, similar to the previous findings, in the transfer payment contract, the freshness preservation effort, the selling price, the freshness of the product, as well as the utility levels of the retailer and the supply chain entity all increase with the government subsidy coefficient. However, there is no direct correlation between the wholesale price and government subsidy. This is because the supplier sells fresh product at the lower wholesale price that they have agreed to in advance under the contract, so the government subsidy does not have an impact on the wholesale price. However, government subsidies still help to enhance the freshness of products and improve the efficiency of the supply chain. Similarly, due to the complexity of the mathematical expressions for the derivative of the transfer payment fee, and the utility level of the supplier with respect to the government subsidy coefficient, the dynamic relationship between these variables will be further explored through simulation analyses subsequently.

## 5. Simulation analysis

This study takes the sales of perch farmed by S Cooperative at B Supermarket as an example. Based on perch sales data from a B supermarket in Changsha, Hunan Province, we define the potential market demand for perch in this region is 2000 units, the price sensitivity coefficient of the market demand is 0.5, the freshness sensitivity coefficient of the product is 0.5, the initial freshness is 0.85, the life cycle is 5 days, and the extreme value of freshness attenuation is 0.8. The relevant parameters are shown in Table 2. Considering the complexity of the expressions derived from the previous analysis, MATLAB is used for numerical analysis of the model. The study investigates the effects of government subsidies, risk aversion, and contractual parameters under four contracts on the optimal decisions of the supply chain. Furthermore, the study conducts a comparative analysis of the utility levels of the supply chain members and the overall system.

**Table 2:** Table of values of relevant parameters.

Parameters	a	b	l	T	t	$\theta_{0}$	$\varphi_{0}$	η	k	$\delta^{2}$
Value	2000	0.5	0.5	5	1	0.85	0.8	0.2	150	1

### 5.1. Analysis of effect of government subsidy, risk aversion and contractual parameters on supply chain decisions

Tables 3–5 discuss the optimal supply chain decisions under different risk aversion levels of the supplier when the government subsidy coefficients are 0.2, 0.5 and 0.8 respectively. From Table 3, it can be observed that the more the S cooperative tends to be risk-averse, that is, the larger λ is, the lower the transfer payment fees required by B cooperative in the transfer payment contract, as well as the wholesale prices and preservation efforts provided by B cooperative in the other three contracts, and the lower the selling price of the perch. Combining Tables 4 and 5, it can be found that the more the government subsidizes the S cooperative in the freshness preservation equipment and facilities, the higher the transfer payment fees required by B cooperative in the transfer payment contract, as well as the wholesale prices and preservation efforts provided by B cooperative in the other three contracts, and the higher the selling price of the perch. In the transfer payment contract and the cost-sharing contract with a high cost-sharing ratio, the freshness preservation effort level of B Cooperative is relatively high.

**Table 3:** When z=0.2, effect of λ,γ,χ on supply chain decisions.

Contracts		λ	w	τ	p	F
PF		0.2	1111.36	0.4631	2556.14	–
		0.4	769.4	0.3206	2385.14	–
		0.6	588.363	0.2452	2296.61	–
CB	γ=0.2	0.2	1111.37	0.5788	2556.15	–
		0.4	769.403	0.4007	2385.15	–
		0.6	588.365	0.3064	2294.62	–
	γ=0.5	0.2	1111.39	0.9262	2556.2	–
		0.4	769.413	0.6412	2385.18	–
		0.6	588.37	0.4903	2294.64	–
	γ=0.8	0.2	1111.47	2.3156	2556.37	–
		0.4	769.45	1.6030	2385.29	–
		0.6	588.392	1.2258	2294.73	–
SY	χ=0.1	0.2	887.285	0.4985	2493.39	–
		0.4	562.531	0.3482	2312.96	–
		0.6	388.275	0.2675	2216.14	–
	χ=0.2	0.2	678.193	0.5385	2424.33	–
		0.4	373.125	0.3796	2233.65	–
		0.6	207.078	0.2931	2129.86	–
	χ=0.3	0.2	486.388	0.5837	2347.89	–
		0.4	203.835	0.4155	2146.05	–
		0.6	47.6885	0.3226	2034.5	–
PZ		0.2	0	1.6672	2000.58	957467
		0.4	0	1.6672	2000.58	695842
		0.6	0	1.6672	2000.58	545503

**Table 4:** When z=0.5, effect of λ,γ,χ on supply chain decisions.

Contracts		λ	w	τ	p	F
PF		0.2	1111.38	0.7409	2556.17	–
		0.4	769.408	0.5129	2385.16	–
		0.6	588.367	0.3922	2294.63	–
CB	γ=0.2	0.2	1111.39	0.9262	2556.2	–
		0.4	769.413	0.6412	2385.18	–
		0.6	588.37	0.4903	2294.64	–
	γ=0.5	0.2	1111.42	1.4819	2556.27	–
		0.4	769.428	1.0259	2385.23	–
		0.6	588.379	0.7845	2294.68	–
	γ=0.8	0.2	1111.54	3.7052	2556.55	–
		0.4	769.487	2.5650	2385.41	–
		0.6	588.413	1.9614	2294.81	–
SY	χ=0.1	0.2	887.298	0.7976	2493.43	–
		0.4	562.537	0.5571	2312.99	–
		0.6	388.278	0.4280	2216.16	–
	χ=0.2	0.2	678.204	0.8615	2424.37	–
		0.4	373.13	0.6073	2233.68	–
		0.6	207.08	0.4689	2129.88	–
	χ=0.3	0.2	486.397	0.9339	2347.93	–
		0.4	203.837	0.6648	2146.07	–
		0.6	47.689	0.5161	2034.52	–
PZ		0.2	0	2.6676	2000.68	957638
		0.4	0	2.6676	2000.68	696017
		0.6	0	2.6676	2000.68	545682

**Table 5:** When z=0.8, effect of λ,γ,χ on supply chain decisions.

Contracts		λ	w	τ	p	F
PF		0.2	1111.44	1.8524	2556.31	–
		0.4	769.437	1.2824	2385.26	–
		0.6	588.384	0.9806	2294.7	–
CB	γ=0.2	0.2	1111.47	2.3156	2556.37	–
		0.4	769.45	1.6030	2385.29	–
		0.6	588.392	1.2258	2294.73	–
	γ=0.5	0.2	1111.54	3.7052	2556.55	–
		0.4	769.487	2.5650	2385.41	–
		0.6	588.413	1.9614	2294.81	–
	γ=0.8	0.2	1111.85	9.2654	2557.26	–
		0.4	769.635	6.4136	2385.87	–
		0.6	588.5	4.9042	2295.15	–
SY	χ=0.1	0.2	887.351	1.9942	2493.58	–
		0.4	562.56	1.3928	2313.08	–
		0.6	388.29	1.0700	2216.23	–
	χ=0.2	0.2	678.248	2.1540	2424.53	–
		0.4	373.147	1.5183	2233.78	–
		0.6	207.087	1.1724	2129.96	–
	χ=0.3	0.2	486.431	2.3350	2348.09	–
		0.4	203.847	1.6622	2146.18	–
		0.6	47.6908	1.2903	2034.6	–
PZ		0.2	0	6.6703	2001.08	958323
		0.4	0	6.6703	2001.08	696718
		0.6	0	6.6703	2001.08	546397

Furthermore, it was also discovered that in the cost-sharing contract, the more B Supermarket shares in the freshness preservation costs for S Cooperative, the higher the wholesale price and preservation efforts provided by S Cooperative will be, and the higher the selling price of the perch will be. However, in the profit-sharing contract, the more sales profits B Supermarket shares with S Cooperative, the lower the wholesale price provided by S Cooperative will be, but its preservation efforts will increase, and the selling price of the perch will decrease.

### 5.2. Comparative analysis of the supply chain members and the overall system utility level

**Fig 3:**
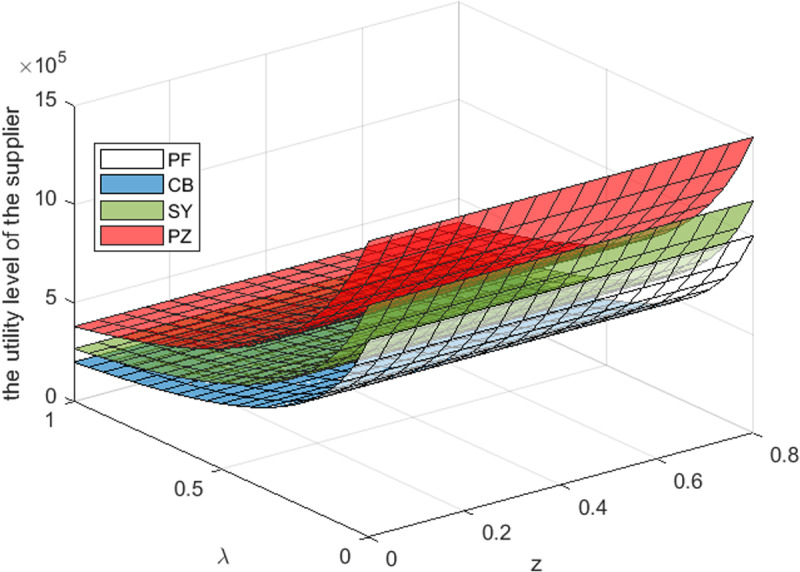
Effect of λ,z **on the utility level of the supplier.**

**Fig 4:**
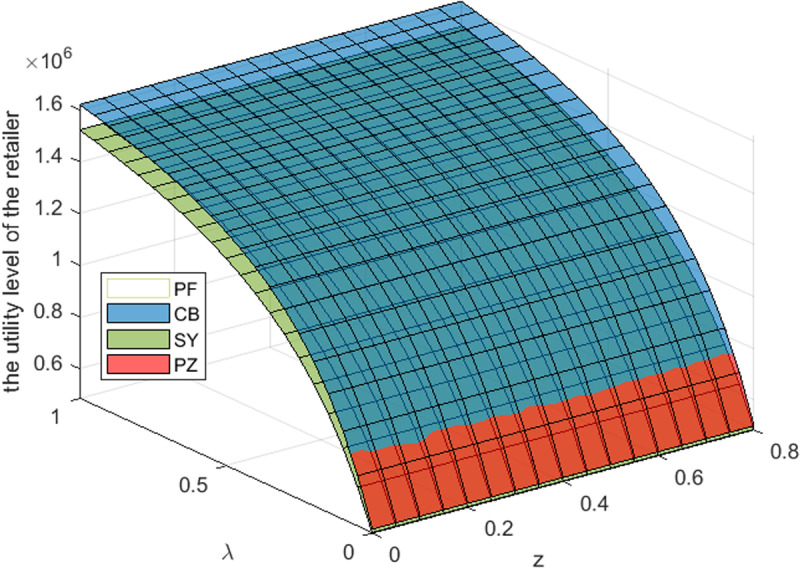
Effect of λ,z **on the utility level of the retailer.**

**Fig 5:**
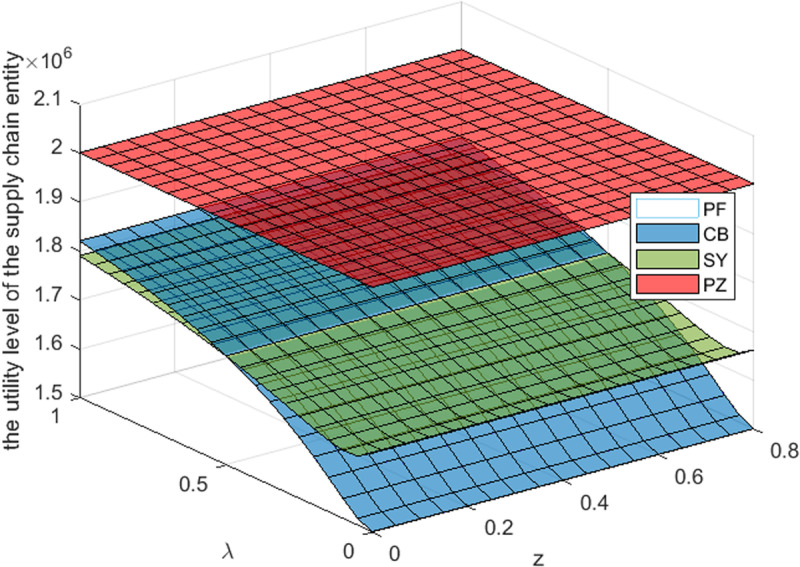
Effect of λ,z **on the utility level of the supply chain entity.**

This section takes the cost-sharing ratio is 0.5, and the revenue-sharing is 0.3. By referring to Figs 3–5– and Tables 6–8, it can be observed that: For S Cooperative, its utility level is the highest under the transfer payment contract. For B Supermarket, when the S Cooperative’s risk aversion degree is lower than a certain threshold, its utility level under the transfer payment contract is the highest. When the S Cooperative’s risk aversion degree exceeds this threshold, its utility level under the cost-sharing contract is the highest. For the entire supply chain, its utility level is the highest under the transfer payment contract. Furthermore, compared with the impact of government subsidies on the utility levels of supply chain members and the entire system, the impact of S Cooperative’s risk aversion on the utility levels of supply chain members and the entire system is more significant_._

**Table 6:** The relationship between λ,z and $U(\pi_{S})$.

	λ=0,z=0	λ=0.8,z=0	λ=0,z=0.8	λ=0.8,z=0.8
$U(\pi_{S}^{PF})$	1000440	238195	1000580	238202
$U(\pi_{S}^{CB})$	1000480	238196	1000740	238212
$U(\pi_{S}^{SY})$	1177000	316388	1177180	316402
$U(\pi_{S}^{PZ})$	1500710	448138	1501110	448573

**Table 7:** The relationship between λ,z and $U(\pi_{R})$.

	λ=0,z=0	λ=0.8,z=0	λ=0,z=0.8	λ=0.8,z=0.8
$U(\pi_{R}^{PF})$	500238	1552810	500371	1552910
$U(\pi_{R}^{CB})$	500205	1552830	500204	1553020
$U(\pi_{R}^{SY})$	484665	1452460	484817	1452580
$U(\pi_{R}^{PZ})$	500238	1552810	500371	1552910

**Table 8:** The relationship between λ,z and $U(\pi_{O})$.

	λ=0,z=0	λ=0.8,z=0	λ=0,z=0.8	λ=0.8,z=0.8
$U(\pi_{O}^{PF})$	1500680	1791010	1500950	1791110
$U(\pi_{O}^{CB})$	1500680	1791030	1500950	1791230
$U(\pi_{O}^{SY})$	1661660	1768840	1662000	1768980
$U(\pi_{O}^{PZ})$	2000950	2000950	2001490	2001490

## 6. Conclusions and recommendations

This paper investigates the fresh product supply chain management decision-making problem under government subsidies and supplier’s risk aversion. By establishing and solving the game models under four different trading contracts, we obtained the optimal decisions of the supply chain, the freshness of products, as well as the utility levels of members and the entire system of the supply chain. Furthermore, we explored the effects of the supplier’s risk-aversion behavior, government subsidies, and contract parameters on the supply chain. The primary conclusions and implications drawn from this study are as follows,

(1) The increase in government subsidies will enhance the utility levels of the supplier, retailer, and the supply chain entity. The more subsidies there are, the more positive the performance of supply chain members and the entire system will be. This research shows that wholesale prices, freshness preservation efforts, product freshness and selling prices in the supply chain all show an upward trend with the increase of government subsidies. In the transfer payment contract, there is no direct correlation between government subsidies and wholesale prices because the supplier has already agreed on lower wholesale prices in the contract.
(2) The degree of risk aversion of the supplier has a significant impact on its decisions within the supply chain. When the supplier tends to be risk-averse, it will reduce transfer payment fees, wholesale prices, and freshness preservation efforts, thereby lowering the product freshness and its own utility levels. At the same time, it will prompt the retailer to lower selling prices, but it helps to enhance the utility levels of both the retailer and the supply chain entity. Compared with government subsidies, the impact of the supplier’s risk-averse behavior on the utility levels of supply chain members and the overall system is more significant.
(3) In the transfer payment contract and the cost-sharing contract with a high cost-sharing ratio, the preservation effort level of the supplier is relatively high. Under the transfer payment contract, the supplier has the highest utility level. When the supplier’s risk aversion degree is below a certain threshold, the retailer has the highest utility level under the transfer payment contract. When the supplier’s risk aversion degree exceeds this threshold, the retailer has the highest utility level under the cost-sharing contract. The supply chain entity has the highest utility level under the transfer payment contract.

Based on the above research conclusions, we can draw the following management implications:

(1) Government subsidies play a positive role in enhancing the effectiveness of supply chain members and the entire system. Therefore, the government can consider increasing subsidies when necessary to promote the healthy development of the supply chain and improve market efficiency. When implementing subsidy policies, the government should pay attention to the specific impacts of subsidies on each link of the supply chain (such as wholesale prices, freshness preservation efforts, and selling prices), ensuring that subsidies can effectively reach consumers and avoiding excessive price hikes in the middle links.
(2) The degree of risk aversion of the supplier has a significant impact on its decisions and the overall utility of the supply chain. When the supplier tends to be risk-averse, although it will reduce its own utility level, it may help to enhance the utility levels of the retailer and the supply chain entity. Therefore, enterprises should pay attention to the risk preferences of the supplier and find a balance point between risk aversion and overall utility. Enterprises can also reduce the degree of risk aversion of the supplier by establishing long-term and stable cooperative relationships with it and providing risk-sharing mechanisms, thereby enhancing the overall utility of the supply chain.
(3) The transfer payment contract demonstrates outstanding performance in enhancing the utility of the supplier, the retailer, and the supply chain entity. Therefore, when choosing supply chain contracts, enterprises can give priority to transfer payment contracts. However, when the risk aversion degree of the supplier exceeds a certain threshold, the utility level of the retailer under cost-sharing contracts may be higher. This indicates that enterprises need to flexibly select contract types based on the risk aversion degree of the supplier.
(4) When managing the supply chain, enterprises should comprehensively consider factors such as government subsidies, the degree of risk aversion of the supplier, and contract selection. They should formulate comprehensive management strategies. Moreover, by strengthening internal communication and collaboration within the supply chain, they can enhance the overall response speed and flexibility of the supply chain to cope with challenges and opportunities in the external environment.

## 7. Limitations

In order to facilitate theoretical analysis and model construction, this paper sets government subsidies as exogenous parameters and assumes that the government is not involved in the decision-making of fresh product supply chain members, but in practice, the formulation of government subsidy policies is often affected by the decision-making behavior of subsidy recipients. In addition, this paper considers the price and freshness input decisions made by the chain members under information symmetry, however, in the real market environment, bilateral information asymmetry is common, which may further complicate the interaction between the supply chain members and their decision-making process. Therefore, the inclusion of the government as one of the decision makers and the examination of supply chain management decisions under information asymmetry could be a direction for further research in the future.

## Supporting information

S1 Fig Two-tier fresh product supply chain mode. (TIF)

S2 Fig Decision sequence for supplier-retailer two-tier fresh product supply chain. (TIF)

S3 FigEffect of λ,z on the utility level of the supplier.(TIF)

S4 FigEffect of λ,z on the utility level of the retailer.(TIF)

S5 FigEffect of λ,z on the utility level of the supply chain entity.(TIF)

S1 Table Symbol description. (XLSX)

S2 Table Table of values of relevant parameters. (XLSX)

S3 TableWhen z=0.2, effect of λ,γ,χ on supply chain decisions.(XLSX)

S4 TableWhen z=0.5, effect of λ,γ,χ on supply chain decisions.(XLSX)

S5 TableWhen z=0.8, effect of λ,γ,χ on supply chain decisions.(XLSX)

S6 TableThe relationship between λ,z and $U(\pi_{S})$.(XLSX)

S7 TableThe relationship between λ,z and $U(\pi_{R})$.(XLSX)

S8 TableThe relationship between λ,z and $U(\pi_{O})$.(XLSX)

S1 Appendix The proof process of the relevant theorems and corollaries. (DOCX)
