# Supplementary figures and images for: A study on fresh product supply chain management decisions considering subsidies and different transaction contracts
Source: PLoS One. 2025 May 29;20(5):e0322800. doi: 10.1371/journal.pone.0322800 (PMC12122046; doi:10.1371/journal.pone.0322800)

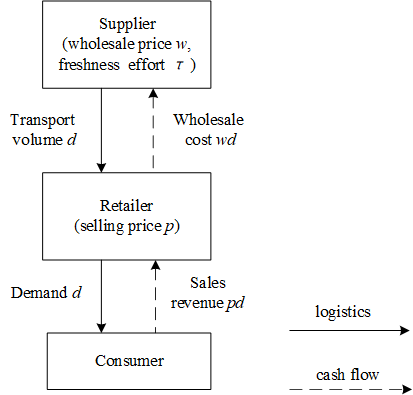

Supplement: S1 Fig — (TIF) [file pone.0322800.s001.tif]

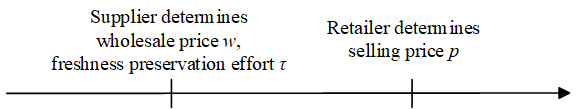

Supplement: S2 Fig — (TIF) [file pone.0322800.s002.tif]

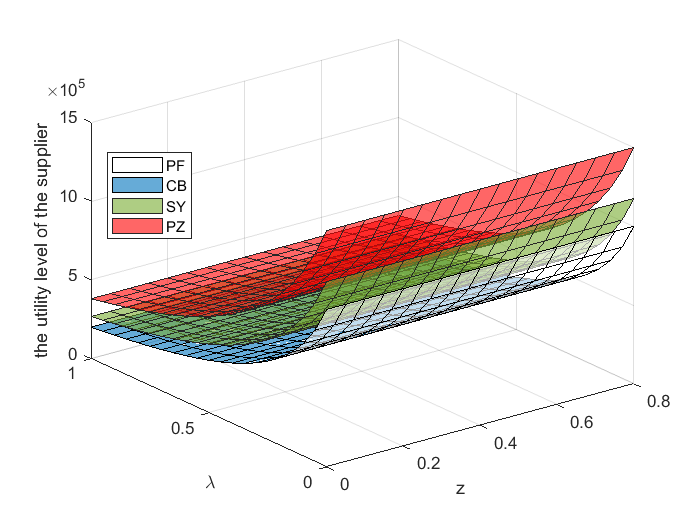

Supplement: S3 Fig — (TIF) [file pone.0322800.s003.tif]

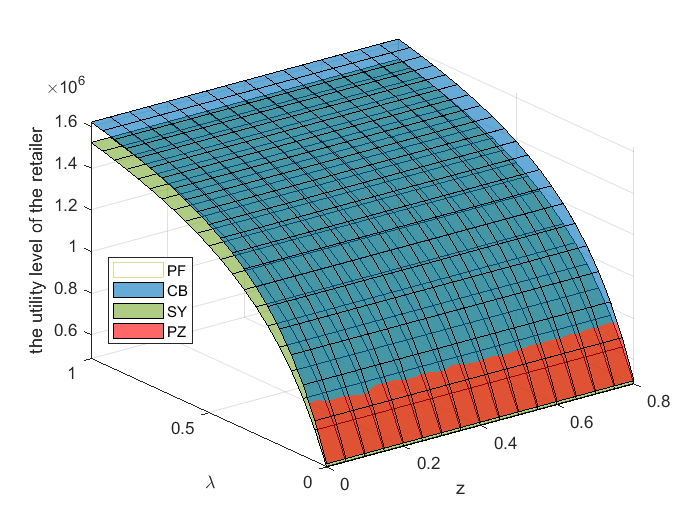

Supplement: S4 Fig — (TIF) [file pone.0322800.s004.tif]

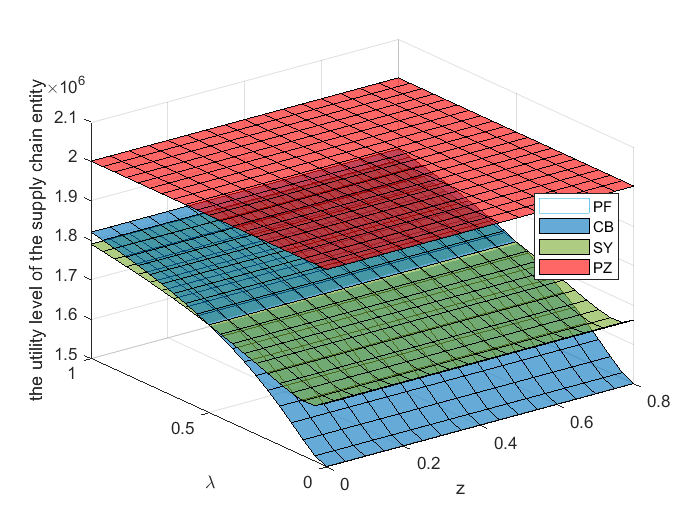

Supplement: S5 Fig — (TIF) [file pone.0322800.s005.tif]
