# Supplementary material for: A study on fresh product supply chain management decisions considering subsidies and different transaction contracts
Source: PLoS One. 2025 May 29;20(5):e0322800. doi: 10.1371/journal.pone.0322800 (PMC12122046; doi:10.1371/journal.pone.0322800)
Supplement: S1 Appendix — (DOCX) [file pone.0322800.s014.docx]

**Appendix**

**Proof of Theorem 1**

According to the inverse induction method, we obtain $\frac{\partial^{2}U(\pi_{R}^{PF})}{\partial p^{2}}=-2b<0$ firstly, that is, the existence of the optimal sales price $p^{PF*}$ such that $U(\pi_{R}^{PF})$ has a maximum value, according to , we get . Substituting $p^{PF*}$ into equation (5), we obtain the Hessian matrix $H^{PF}=\left[\begin{matrix} -b-2\lambda\delta^{2} & \frac{l\eta}{2} \\ \frac{l\eta}{2} & -k(1-z) \end{matrix}\right]$ , from $-b-2\lambda\delta^{2}<0$, we can know that when $-\frac{1}{4}l^{2}\eta^{2}-k(1-z)(-b-2\lambda\delta^{2})>0$, $H^{PF}$ is a negative definite matrix, and can be solved by $\frac{\partial U(\pi_{S}^{PF})}{\partial w}=0$and $\frac{\partial U(\pi_{S}^{PF})}{\partial\tau}=0$, then we obtain , .

We substitute the value of $w^{PF*},\tau^{PF*}$ in $p^{PF*}$, then we get , this leads to the market demand, supplier and seller utility levels respectively, , , .

**Proof of Corollary 1**

Due to $-b-2\lambda\delta^{2}<0$, $l^{2}\eta^{2}+4k(-1+z)(b+2\lambda\delta^{2})<0$, , we can get,

,

,

,

,

,

When $0<k<\frac{l^{2}\eta^{2}}{2b\text{（}1-z\text{）}}$, $\frac{\partial U(\pi_{R}^{PF*})}{\partial\lambda}<0$, $\frac{\partial U(\pi_{O}^{PF*})}{\partial\lambda}<0$; when $\frac{l^{2}\eta^{2}}{2b\text{（}1-z\text{）}}<k<-\frac{l^{2}\eta^{2}(b+8\lambda\delta^{2})}{8b(-1+z)\lambda\delta^{2}}$, $\frac{\partial U(\pi_{R}^{PF*})}{\partial\lambda}>0$, $\frac{\partial U(\pi_{O}^{PF*})}{\partial\lambda}<0$; when $k>-\frac{l^{2}\eta^{2}(b+8\lambda\delta^{2})}{8b(-1+z)\lambda\delta^{2}}$, $\frac{\partial U(\pi_{R}^{PF*})}{\partial\lambda}>0$, $\frac{\partial U(\pi_{O}^{PF*})}{\partial\lambda}>0$.

**Proof of Corollary 2**

,

**Proof of Theorem 2**

By $\frac{\partial^{2}U(\pi_{R}^{CB})}{\partial p^{2}}=-2b<0$ can be seen that $U(\pi_{R}^{CB})$ is a concave function about $p$ and has a unique maximum value, so from , there is a unique optimal solution for the selling price of fresh products. Substituting $p^{CB*}$ into Eq. (7), the Hessian matrix $H^{CB}=\left[\begin{matrix} -b-2\lambda\delta^{2} & \frac{l\eta}{2} \\ \frac{l\eta}{2} & -k(1-z)(1-\gamma) \end{matrix}\right]$ is obtained. It is known that the first-order principal divisor $D_{1}^{CB}=-b-2\lambda\delta^{2}<0$, when the second-order principal divisor $D_{2}^{CB}=-\frac{1}{4}l^{2}\eta^{2}+k(-1+z)(-1+\gamma)(b+2\lambda\delta^{2})>0$, $H^{CB}$ is a negative definite matrix , so $w^{CB},\tau^{CB}$ are the only optimal solution. Therefore, by $\frac{\partial U(\pi_{S}^{CB})}{\partial w}=0\text{，}\frac{\partial U(\pi_{S}^{CB})}{\partial\tau}=0$, we obtain , . We substitute the value of $w^{CB*},\tau^{CB*}$ in $p^{CB*}$, then we get , this leads to the market demand, supplier and seller utility levels respectively, ,

**Proof of Corollary 3**

Due to $-b-2\lambda\delta^{2}<0$, $l^{2}\eta^{2}-4k(-1+z)(-1+\gamma)(b+2\lambda\delta^{2})<0$, , and $2k(-1+z)(-1+\gamma)^{2}(b^{2}+8\lambda\delta^{2}(b+2\lambda\delta^{2}))+bl^{2}\eta^{2}\gamma<0$, we can get,

,

,

,

,

**Proof of Corollary 4**

,

,

,

,

**Proof of Corollary 5**

,

,

,

,

**Proof of Theorem 3**

By $\frac{\partial^{2}U(\pi_{R}^{SY})}{\partial p^{2}}=-2b(1-\chi)<0$ can be seen that $U(\pi_{R}^{SY})$ is a concave function about $p$ and has a unique maximum value, so from , there is a unique optimal solution for the selling price of fresh products. Substituting $p^{SY*}$ into Eq. (11), the Hessian matrix $H^{SY}=\left[\begin{matrix} \frac{(b-(-2+\chi)\lambda\delta^{2})(-2+\chi)}{2(-1+\chi)^{2}} & \frac{l\eta(b(-1+\chi)-\lambda\delta^{2}(-2+\chi)\chi)}{2b(-1+\chi)} \\ \frac{l\eta(b(-1+\chi)-\lambda\delta^{2}(-2+\chi)\chi)}{2b(-1+\chi)} & \frac{2b^{2}k(-1+z)+l^{2}\eta^{2}\chi(b-\lambda\delta^{2}\chi)}{2b^{2}} \end{matrix}\right]$ is obtained. It is known that the first-order principal divisor $D_{1}^{SY}=\frac{(b-(-2+\chi)\lambda\delta^{2})(-2+\chi)}{2(-1+\chi)^{2}}<0$, when the second-order principal divisor $D_{2}^{SY}=-\frac{l^{2}\eta^{2}-2bk(-1+z)(-2+\chi)+2k(-1+z)\lambda\delta^{2}(-2+\chi)^{2}}{4(-1+\chi)^{2}}>0$, $H^{SY}$ is a negative definite matrix , so $w^{SY},\tau^{SY}$ are the only optimal solution. Therefore, by $\frac{\partial U(\pi_{S}^{SY})}{\partial w}=0\text{，}\frac{\partial U(\pi_{S}^{SY})}{\partial\tau}=0$, we obtain , . We substitute the value of $w^{SY*},\tau^{SY*}$ in $p^{SY*}$, then we get , this leads to the market demand, supplier and seller utility levels respectively, , .

**Proof of Corollary 6**

Due to $b-(-2+\chi)\lambda\delta^{2}>0$, $l^{2}\eta^{2}+2k(-1+z)(-2+\chi)(-b+\lambda\delta^{2}(-2+\chi))<0$, thus

,

,

When $0<k<\frac{l^{2}\eta^{2}\chi}{2b(-1+z)(-2+\chi)}$, $\frac{\partial w^{SY*}}{\partial\lambda}>0$, $\frac{\partial U(\pi_{R}^{SY*})}{\partial\lambda}<0$; when $\frac{l^{2}\eta^{2}\chi}{2b(-1+z)(-2+\chi)}<k<\frac{l^{2}\eta^{2}}{b(-1+z)(-2+\chi)}$, $\frac{\partial w^{SY*}}{\partial\lambda}<0$, $\frac{\partial U(\pi_{R}^{SY*})}{\partial\lambda}<0$; when $k>\frac{l^{2}\eta^{2}}{b(-1+z)(-2+\chi)}$, $\frac{\partial w^{SY*}}{\partial\lambda}<0$, $\frac{\partial U(\pi_{R}^{SY*})}{\partial\lambda}>0$.

**Proof of Corollary 7**

Due to $b-(-2+\chi)\lambda\delta^{2}>0$, $l^{2}\eta^{2}+2k(-1+z)(-2+\chi)(-b+\lambda\delta^{2}(-2+\chi))<0$, $b-b\chi+\lambda\delta^{2}(-2+\chi)\chi>0$, $b(-3+2\chi)-2\lambda\delta^{2}(-2+\chi)(-1+\chi)>0$, thus

**Proof of Corollary 8**

**Proof of Theorem 4**

By $\frac{\partial^{2}U(\pi_{R}^{PZ})}{\partial p^{2}}=-2b<0$ can be seen that $U(\pi_{R}^{PZ})$ is a concave function about $p$ and has a unique maximum value, so from , there is a unique optimal solution for the selling price of fresh products. Substituting $p^{PZ*}$ into Eq. (14), we obtain .

By $U(\pi_{R}^{PZ*})=U(\pi_{R}^{PF*})$, The boundary conditions under which the seller pays the transfer fee can be found, that is .

We substitute the value of $p^{SY*}\text{,}F$ in Eq. (13), and make $\frac{\partial U(\pi_{S}^{PZ})}{\partial w}=0\text{，}\frac{\partial U(\pi_{S}^{PZ})}{\partial\tau}=0$, then we obtain the optimal wholesale price and the level of preservation effort of the supplier are obtained as . Substituting the value of $w^{PZ*},\tau^{PZ*}$ in $p^{SY*},F$, we obtain , . At this point the market demand, the members of the fresh food supply chain and the overall utility are,

,

,

,

.

**Proof of Corollary 9**

Due to $2bk(-1+z)+l^{2}\eta^{2}<0$, $4bk(-1+z)+l^{2}\eta^{2}+8k(-1+z)\lambda\delta^{2}<0$, we can obtain,

$\frac{\partial w^{PZ*}}{\partial\lambda}=0$, $\frac{\partial\tau^{PZ*}}{\partial\lambda}=0$, $\frac{\partial p^{PZ*}}{\partial\lambda}=0$, $\frac{\partial\theta^{PZ*}}{\partial\lambda}=0$, $\frac{\partial U(\pi_{O}^{PZ*})}{\partial\lambda}=0$,

,

,

**Proof of Corollary 10**

$\frac{\partial w^{PZ*}}{\partial z}=0$, ,

, ,
